# Conversion Surgery Following Immunochemotherapy in Initially Unresectable Locally Advanced Esophageal Squamous Cell Carcinoma—A Real-World Multicenter Study (RICE-Retro)

**DOI:** 10.3389/fimmu.2022.935374

**Published:** 2022-07-13

**Authors:** Shujie Huang, Hansheng Wu, Chao Cheng, Ming Zhou, Enwu Xu, Wanli Lin, Guangsuo Wang, Jiming Tang, Xiaosong Ben, Dongkun Zhang, Liang Xie, Haiyu Zhou, Gang Chen, Weitao Zhuang, Yong Tang, Fangping Xu, Zesen Du, Zefeng Xie, Feixiang Wang, Zhe He, Hai Zhang, Xuefeng Sun, Zijun Li, Taotao Sun, Jianhua Liu, Shuhan Yang, Songxi Xie, Junhui Fu, Guibin Qiao

**Affiliations:** ^1^ Department of Thoracic Surgery, Guangdong Provincial People’s Hospital, Guangdong Academy of Medical Sciences, Guangzhou, China; ^2^ Shantou University Medical College, Shantou, China; ^3^ Department of Thoracic Surgery, The First Affiliated Hospital of Shantou University Medical College, Shantou, China; ^4^ Department of Thoracic Surgery, The First Affiliated Hospital of Sun Yat-sen University, Guangzhou, China; ^5^ Department of Thoracic Surgery, The Affiliated Cancer Hospital of Guangzhou Medical University, Guangzhou, China; ^6^ Department of Thoracic Surgery, General Hospital of Southern Theater Command, PLA, Guangzhou, China; ^7^ Department of Thoracic Surgery, Gaozhou People’s Hospital, Gaozhou, China; ^8^ Department of Thoracic Surgery, Shenzhen Institute of Respiratory Disease, Shenzhen People’s Hospital, The Second Clinical Medical College, Jinan University, The First Affiliated Hospital, Southern University of Science and Technology, Shenzhen, China; ^9^ Department of Pathology and Laboratory Medicine, Guangdong Provincial People’s Hospital, Guangdong Academy of Medical Sciences, Guangzhou, China; ^10^ Department of Surgical Oncology, Shantou Central Hospital, Shantou, China; ^11^ Department of General Practice, Guangdong Provincial People’s Hospital, Guangdong Academy of Medical Sciences, Guangzhou, China; ^12^ Guangdong Provincial Geriatrics Institute, Guangdong Provincial People’s Hospital, Guangdong Academy of Medical Sciences, Guangzhou, China; ^13^ WeiLun PET Center, Department of Nuclear Medicine, Guangdong Provincial People’s Hospital, Guangdong Academy of Medical Sciences, Guangzhou, China; ^14^ Department of Oncology, Guangdong Provincial People’s Hospital, Guangdong Academy of Medical Sciences, Guangzhou, China; ^15^ Chronic Disease Laboratory, School of Medicine, South China University of Technology, Guangzhou, China; ^16^ Department of Radiation Oncology, Guangdong Provincial People’s Hospital, Guangdong Academy of Medical Sciences, Guangzhou, China; ^17^ The Second School of Clinical Medicine, Southern Medical University, Guangzhou, China

**Keywords:** esophageal squamous cell carcinoma, conversion surgery, immunotherapy, effectiveness, real-world study

## Abstract

**Purpose:**

The present study sets out to evaluate the feasibility, safety, and effectiveness of conversion surgery following induction immunochemotherapy for patients with initially unresectable locally advanced esophageal squamous cell carcinoma (ESCC) in a real-world scenario.

**Materials and Methods:**

In this multi-center, real-world study (NCT04822103), patients who had unresectable ESCC disease were enrolled across eight medical centers in China. All patients received programmed death receptor-1 (PD-1) inhibitor plus chemotherapy every 3 weeks for at least two cycles. Patients with significant relief of cancer-related clinical symptoms and radiological responsive disease were deemed surgical candidates. Feasibility and safety profile of immunochemotherapy plus conversion surgery, radiological and pathological tumor responses, as well as short-term survival outcomes were evaluated. Moreover, data of an independent ESCC cohort receiving induction chemotherapy (iC) were compared.

**Results:**

One hundred and fifty-five patients were enrolled in the final analysis. Esophagectomy was offered to 116 patients, yielding a conversion rate of 74.8%. R0 resection rate was 94%. Among the 155 patients, 107 (69.0%) patients experienced at least one treatment-related adverse event (TRAE) and 45 (29.0%) patients reported grade 3 and above TRAEs. Significant differences in responsive disease rate were observed between iC cohort and induction immunochemotherapy (iIC) cohort [objective response rate: iIC: 63.2% vs. iC: 47.7%, p = 0.004; pathological complete response: iIC: 22.4% vs. iC: 6.7%, p = 0.001). Higher anastomosis fistula rate was observed in the iC group (19.2%) compared with the iIC group (4%). Furthermore, Significantly higher event-free survival was observed in those who underwent conversion surgery.

**Conclusion:**

Our results supported that conversion surgery following immunochemotherapy is feasible and safe for patients with initially unresectable locally advanced ESCC. Both radiological and pathological response rates were significantly higher in the iIC cohort compared with those in the traditional iC cohort.

## Introduction

Esophageal squamous cell carcinoma (ESCC) could easily penetrate the esophageal wall and invade adjacent organs due to the lack of serosa (1). According to the National Comprehensive Cancer Network guideline, cT4b tumors with evident involvement of the adjacent organs (aorta, trachea, or bronchus) or had multi-station, bulky lymphadenopathy are considered unresectable (ESOPH-C, 1 of 3) (2). The current standard of care for the unresectable locally advanced ESCC is definitive chemoradiation or systemic chemotherapy alone (if local therapy is not indicated) (2); however, the treatment outcomes remain dismal (3). Limited progress has been made in treating unresectable locally advanced ESCC. Thus, novel effective therapeutics are needed.

The emergence of immune checkpoint inhibitors (ICIs) has revolutionized the treatment of advanced or metastatic gastroesophageal cancers (4–7). Recently, the largest randomized, placebo-controlled, phase 3 study (KEYNOTE-590) to date had confirmed better survival benefits of pembrolizumab plus chemotherapy over placebo plus chemotherapy in 749 patients with unresectable locally advanced or metastatic EC. The combination of ICIs and chemotherapy also demonstrated a comparable safety profile to chemotherapy alone (≥G3 TRAEs, 72% vs. 68%) (6). Because of the exciting results released by these clinical trials, NCCN recommended immunotherapy combined with chemotherapy as the first-line treatment for both unresectable locally advanced and metastatic disease (ESOPH-F, 3 of 17) (2). Further, Fan et al. reported that the initially unresectable locally advanced ESCC could be transformed into surgical candidates after receiving immunochemotherapy, and the conversion rate reached 75% (8). Furthermore, recent studies showed that patients receiving induction chemoradiotherapy or chemotherapy followed by conversion surgery could have a better prognosis than those without surgery (9). However, currently, there lacks strong evidence to support the application of conversion surgery following immunochemotherapy in initially unresectable locally advanced ESCC. Hence, this study aimed to evaluate the feasibility, safety, and effectiveness of conversion surgery following induction immunochemotherapy (iIC) for initially unresectable ESCC in a real-world scenario.

## Materials and methods

### Study Design and Participants

The study was designed to be a multi-center and real-world retrospective study (RICE-retro, real-world study of ICI and chemotherapy for advanced esophageal cancer) to investigate the feasibility, safety, and effectiveness of induction ICIs plus chemotherapy at the Guangdong Provincial People’s Hospital, the First Affiliated Hospital of Shantou University Medical College, the First Affiliated Hospital of Sun Yat-sen University, the Affiliated Cancer Hospital of Guangzhou Medical University, General Hospital of Southern Theater Command, Gaozhou People’s Hospital, Shenzhen People’s Hospital and Shantou Central Hospital. The study protocol was reviewed and approved by the Institutional Review Board (IRB) at each participating institution and registered at ClinicalTrials.gov (NCT04822103). Eligible patients were at least 18 years old with an endoscopy-guided, histologically confirmed ESCC, who were deemed unsuitable radiotherapy candidates by radiation oncologists and have radiologically confirmed unresectable cT4b tumors with evident involvement of the adjacent organs (aorta, trachea, or bronchus) or had multi-station, bulky lymphadenopathy before treatment. Confirmed diagnosis of organ invasions was based on the previously reported criteria (1, 10). All patients were treatment-naive, with a Karnofsky Performance Scale (KPS) ≥ 80, adequate organ function, and no distant metastasis. Patients who had previously participated in other interventional clinical trials during their preoperative treatment were excluded from this study. Before the initiation of iIC, all patients received the endoscopy-guided biopsy and contrast-enhanced positron emission tomography (PET)/computed tomography (CT) for diagnostic workup. The clinical and pathologic staging were determined by the surgeons, radiologists and pathologists based on the eight edition staging system of the Union for International Cancer Control/American Joint Committee on Cancer (UICC/AJCC). Baseline measurement of tumor lesions and lymph nodes was based on the Response Evaluation Criteria in Solid Tumors (RECIST) version 1.1 (11).

### Treatment Regimen

The ICIs administered in the current study were PD-1 inhibitors (camrelizumab, pembrolizumab, sintilimab, tislelizumab, toripalimab, and nivolumab), which were administered intravenously at a fixed dose of 200 mg every 3 weeks. The chemotherapy regimen included platinum-based plus docetaxel- or taxane-based agents every 3 weeks intravenously with their doses adjusted by patients’ general condition and the liver or renal functions. All participants enrolled were fully informed of all alternative regimens and provided written consents.

Three to four weeks after the completion of at least two cycles of iIC, contrast-enhanced thoracoabdominal CT or PET/CT was performed for disease evaluation. Tumor responses were denoted by complete response (CR), partial response (PR), stable disease (SD), and progressive disease (PD).

**Figure 1:**
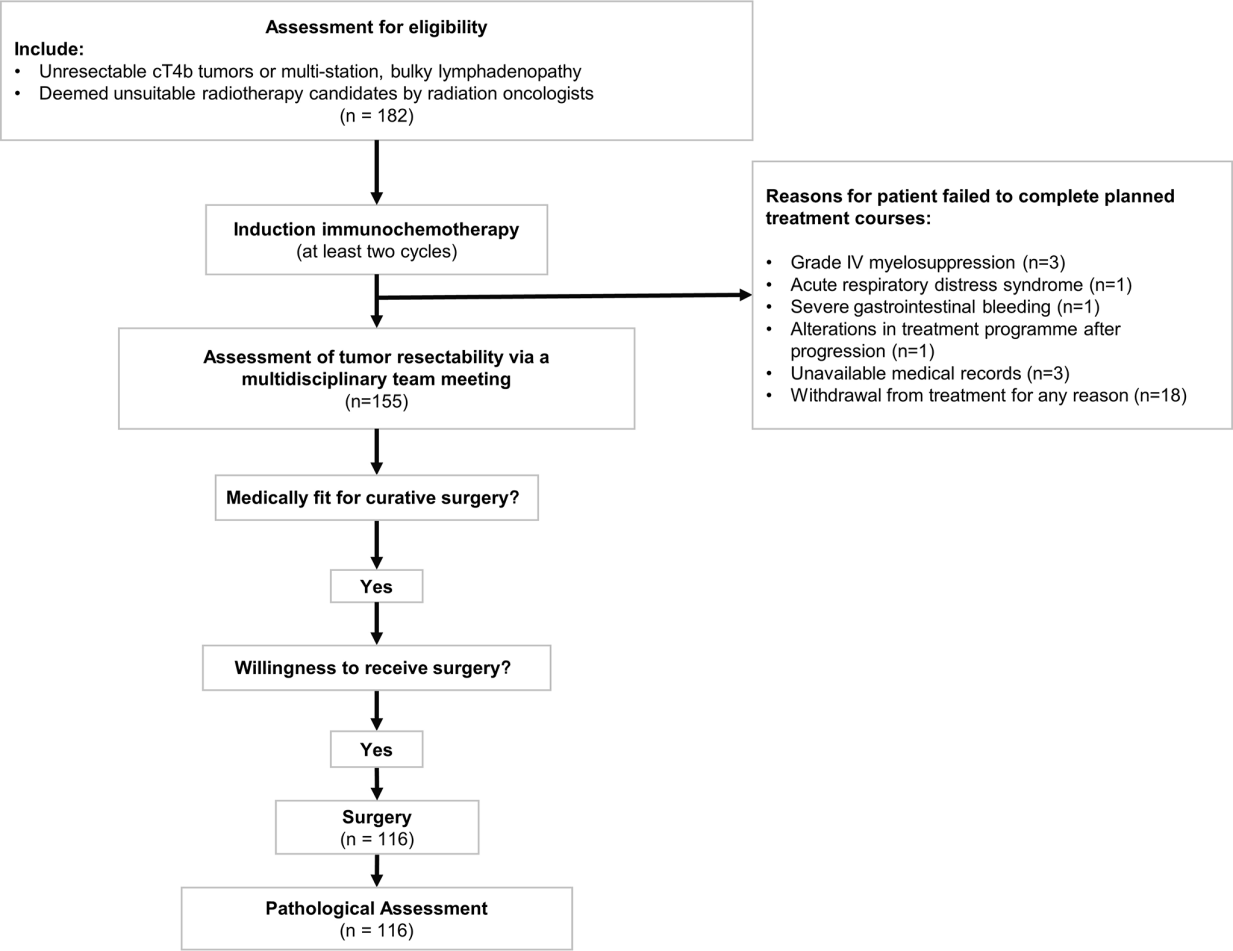
Flowchart of the induction treatment course for initially unresectable esophageal squamous cell carcinoma patients. Patients who met the inclusion criteria received at least two cycles of immunochemotherapy. Patients who failed to complete planned cycles of treatment were excluded from the subsequent analysis. Assessment of tumor response was conducted *via* a multidisciplinary team meeting. McKeown esophagectomy and Ivor Lewis esophagectomy would be performed on medically fit individuals with willingness to receive surgery.

A multidisciplinary team meeting was held during each patient’s radiological evaluation of tumor response. In general, patients with significant relief of cancer-related clinical symptoms and radiological CR/PR diseases were deemed surgical candidates. For patients whose condition was evaluated as SD status, conversion surgery would be performed only if the shrinkage extent of both primary tumor and lymph nodes enables the formation of clear tumor-and-adjacent organ boundary. Furthermore, surgery was deemed unsuitable for those with radiologically confirmed PD. Flowchart of the study design was presented in **Figure 1**.

### Surgery and Pathological Assessments

Minimally invasive esophagectomy with two-field or three-field lymphadenectomy was performed on medically fit patients. McKeown and Ivor Lewis esophagectomy were the two primary surgical approaches. The pathological examination was performed and re-evaluated by two pathologists independently according to the standardized pathological assessment protocol adopted by all research centers to minimize the interobserver variability. Tumor regression grade (TRG) was calculated according to Becker system, a four-tier scoring system estimating the percentage of residual tumor in relationship to the macroscopically identifiable tumor bed (12). Immunohistochemistry staining was performed for PD-1 (clone: MRQ-22, Abcam, 1:50) and PD-L1 (clone: 22C3, Abcam, 1:500), with their expression levels presented as the combined positive score (CPS). CPS was defined as the number of PD-L1 staining cells (tumor cell, lymphocytes, and macrophages) divided by total number of viable tumor cells, multiplied by 100 (13).

### Outcome Evaluation

Feasibility of iIC was defined as at least 80% of the patients completed all planned courses of iIC. Feasibility of conversion surgery was defined as at least 80% of the patients were medically fit for surgery after completion of iIC. Objective response rate (ORR) was defined as best overall response of complete or PR rate, per RECIST version 1.1. The safety profile was assessed by the proportion of participants with ≥ grade 3 adverse events as defined by Common Terminology Criteria for Adverse Events (CTCAE) version 5.0 (14). Confirmation of the relationship between AEs and the drugs in use was based on the WHO-UMC Causality Categories (15). The key secondary end point was pathological CR (pCR) defined as the absence of invasive/*in situ* cancer in the primary lesion site. Major pathological response (mPR) was defined as ≤10% residual viable tumor following iIC (16). R0 resection was defined as the rate of negative margins microscopically (including circumferential resection margin). Event-free survival (EFS) was calculated from the date of treatment initiation to the date of first progression (local recurrence of tumor or distant metastasis) or death from any cause (17). Patients who were lost to follow up or still alive at the time of final analysis were classified as censored data. Downstaging of primary tumor, nodal, or combined TNM stage was recorded if the stage obtained from the pathological examination was earlier than the pretreatment clinical stage (18).

Furthermore, to compare the oncological outcomes of RICE-retro with conventional preoperative chemotherapy, data of a cohort of patients with ESCC receiving induction chemotherapy (iC) from these centers were retrospectively analyzed.

### Statistical Analysis

Descriptive data were reported as mean ± standard deviation (SD), median [interquartile range (IQR)], or frequency (percentage). Comparisons of continuous variables were performed using the Student’s t-test or the Wilcoxon rank sum test as appropriate. Categorical clinicopathological variables were compared by using the Chi-square test or Fisher’s exact test. Two-sided P < 0.05 was considered statistically significant in all tests. All statistical analyses were performed using the software “Statistical Package for Social Science” (SPSS) version 26 for Windows (SPSS, Inc., Chicago, Illinois) and R 4.0.0 (R Core Team 2020) (19). High-quality figures were generated using the R packages.

## Results

### Clinicopathological *Characteristics*

From November 2019 to June 2021, 182 patients with ESCC were included at eight institutions in China and finally 155 patients completed the planned treatment courses. The date of the last follow-up was October 1, 2021. Most patients were male (121 of 155, 78.1%), and the median age was 61 years (IQR, 55–66 years). Baseline clinicopathologic information was presented in **Table 1**. Most tumors were located in the middle (48.4%) and lower (39.4%) portion of the thoracic esophagus. Twenty-one patients had clinical stage III disease before surgery, whereas stage IV disease accounted for 86.5% (n = 134) of patients.

**Table 1:** Clinicopathological characteristics of the RICE cohort.

Characteristics	No. (%)
Sex	
Male	121 (78.1)
Female	34 (21.9)
Age (years)	
Median	61
IQR	55-66
KPS	
80	14 (9.0)
90	141 (91.0)
History of smoking	
Yes	85 (54.8)
No	70 (45.2)
History of drinking	
Yes	63 (40.6)
No	92 (59.4)
Family oncological history	
Yes	33 (21.3)
No	122 (78.7)
Tumor location	
Thoracic upper portion	19 (12.3)
Thoracic middle portion	75 (48.4)
Thoracic lower portion	61 (39.4)
cT	
cT2	1 (0.6)
cT3	24 (15.5)
cT4a	28 (18.1)
cT4b	102 (65.8)
cN	
cN0	60 (38.7)
cN1	65 (41.9)
cN2	24 (15.5)
cN3	6 (3.9)
cTNM	
III	21 (13.5)
IVA	134 (86.5)
pT	
pT0	26 (22.4)
pTis	11 (9.5)
pT1a	10 (8.6)
pT1b	20 (17.2)
pT2	17 (14.7)
pT3	32 (27.6)
pN	
pN0	84 (72.4)
pN1	21 (18.1)
pN2	10 (8.6)
pN3	1 (0.9)
pTNM	
I	68 (58.6)
II	16 (13.8)
IIIA	13 (11.2)
IIIB	18 (15.5)
IVA	1 (0.9)
Lymphovascular invasion	
Yes	9 (7.8)
No	107 (92.2)
Perineureal invasion	
Yes	9 (7.8)
No	107 (92.2)
R0	
R0	104 (94)
R1	7 (6)

Variables are described as n(%) or median [interquartile range (IQR)]. cT, clinical tumor stage; cN, clinical nodal stage; cTNM, clinical tumor-nodal-metastatic stage; pT, pathological tumor stage; pN, pathological nodal stage; pTNM, pathological tumor-nodal-metastatic stage.

### Feasibility

The proportion of patients with successful completion of planned treatment course was 85.2% (155 of 182). Patients failed to complete induction treatment were due to grade IV myelosuppression (3 of 182, 1.6%), acute respiratory distress syndrome (1 of 182, 0.5%), severe gastrointestinal bleeding (1 of 182, 0.5%), and alterations in treatment program after progression (1 of 182, 0.5%). Other patients were excluded from final analysis due to unavailable medical records that would hamper statistical analysis (3 of 182, 1.6%) and withdrawal from treatment for any reason (18 of 182, 9.9%).

Upon the completion of induction treatment, 126 of 155 (81.3%) patients were considered suitable for conversion surgery. Ten patients were unwilling to undergo surgery. Finally, esophagectomy was then offered to the remaining 116 patients, yielding a conversion rate of 74.8%.

### Safety

Among the 155 patients, 107 (69.0%) patients experienced at least one treatment-related adverse event (TRAE) and the common TRAEs included fatigue (80 of 155, 51.6%), nausea (64 of 155, 41.3%), and diarrhea (47 of 155, 30.3%). Grade 3 and above (grade ≥ 3) TRAEs were found by 29.0% (45 of 155) of the patients, including leukopenia (20 of 155, 12.9%), neutropenia (18 of 155, 11.6%), rash (12 of 155, 7.7%), diarrhea (6 of 155, 3.9%), and infection (6 of 155, 3.9%). There were immune-related skin toxicities, including pruritus (47 of 155, 30.3%) and rash (44 of 155, 28%) of any grade. The details of TRAEs observed in our study cohort were shown in **Table 2**, and a clinical heatmap was used to depict the association between clinicopathological characteristics such as radiological tumor response and each type of adverse event (**Supplementary Figure 1**).

**Table 2:** Adverse events during immunochemotherapy and after surgery.

Event	No. (%)
Events of any grade during immunochemotherapy	
Nausea	64 (41)
Vomiting	38 (25)
Diarrhea	47 (30)
Constipation	24 (15)
Dyspnea	15 (10)
Rash	44 (28)
Pruritus	47 (30)
Infection	11 (7)
Pain	39 (25)
Fatigue	80 (52)
Leukopenia	33 (21)
Neutropenia	32 (21)
Lymphopenia	14 (9)
Anemia	21 (14)
Thrombocytopenia	5 (3)
Events of grade ≥ 3 during immunochemotherapy	
Nausea	5 (3)
Vomiting	4 (3)
Diarrhea	6 (4)
Constipation	0 (0)
Dyspnea	1 (1)
Rash	12 (8)
Pruritus	3 (2)
Infection	6 (4)
Pain	2 (1)
Fatigue	3 (2)
Leukopenia	20 (13)
Neutropenia	18 (12)
Lymphopenia	3 (2)
Anemia	3 (2)
Thrombocytopenia	0 (0)
Postoperative events	
Heart issues	3 (3)
Pneumonia	10 (9)
Atelectasis	10 (9)
Pleural effusion	8 (7)
Anastomosis fistula	5 (4)
Wound infection	2 (2)
Hoarseness	2 (2)
Hypoxia	2 (2)
Dysphagia	0 (0)
Hemothorax	0 (0)
Chylothorax	0 (0)
Mediastinitis	0 (0)
Death	1 (1)

The median postoperative time length of hospital stay (PLOS) was 11 (IQR, 8–14) days, and the median operative time was 325 min (IQR, 260–390). Intraoperative blood loss was 100 ml (IQR, 50–100). Postoperative complications are summarized in **Table 2**. Of the 116 patients, five patients (4%) experienced anastomosis fistula, and one patient died within 30 days after surgery.

Significant differences in mean PLOS (iC vs. iIC: 18 ± 14 days vs. 12 ± 9 days, p = 0.005), mean operative time (iC vs. iIC: 395 ± 109 min vs. 332 ± 87 min, p = 0.023), and mean intraoperative blood loss (iC vs. iIC: 199 ± 156 ml vs. 110 ± 88 ml, p = 0.001) were observed between the iC and iIC groups. Higher anastomosis fistula rate was observed in the iC group (19.2%) compared with the iIC group (4%). The details of postoperative events of iC group are provided in **Supplementary Table 1**.

### Effectiveness

**Figure 2:**
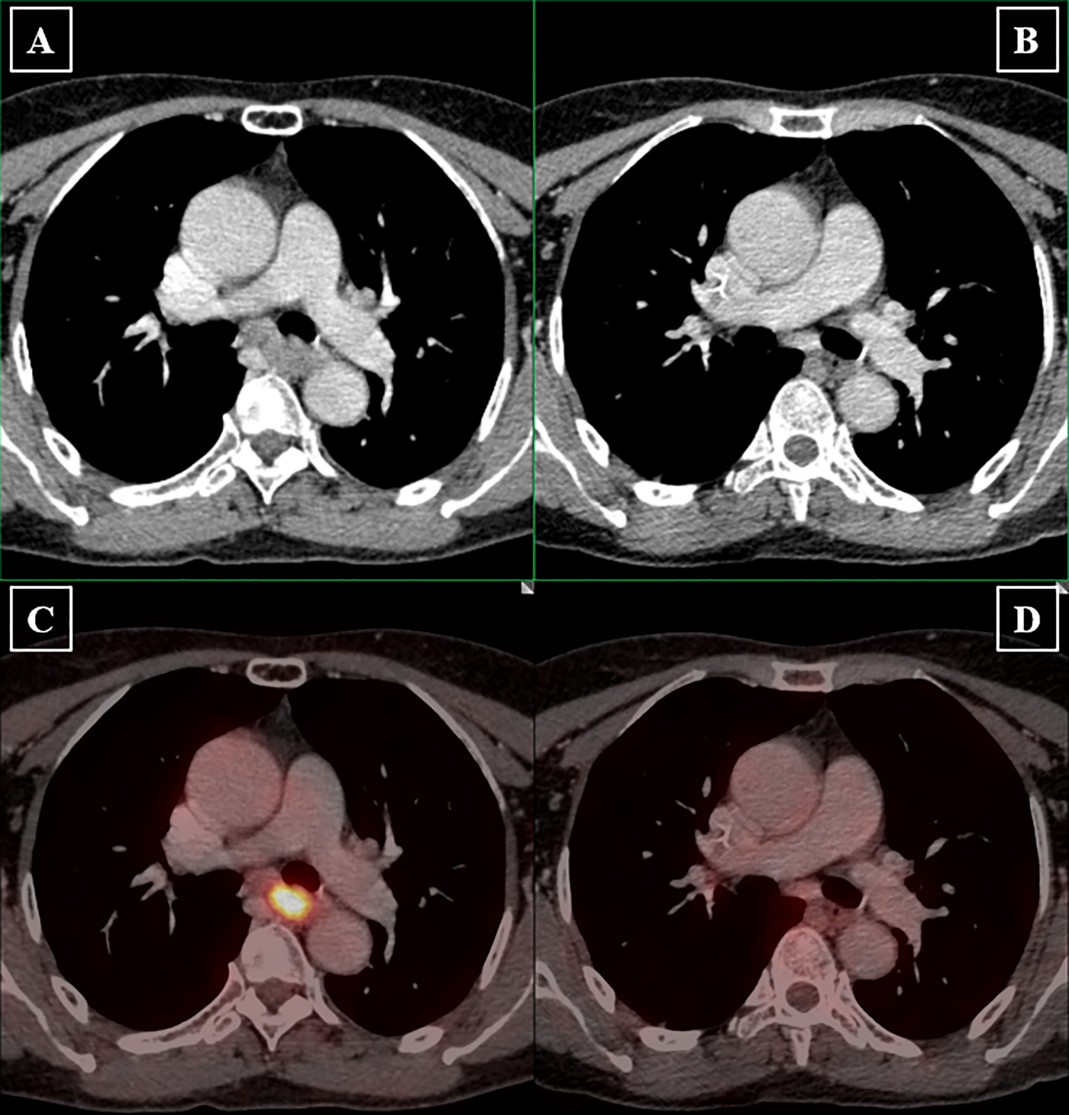
Radiological assessment before and after induction immunochemotherapy. Longest diameters in the plane of measurement of primary lesions were recorded. Lymph nodes were considered malignant if the short axis is longer than 1.5 mm. Pretreatment clinical staging of primary tumor and lymph nodes were determined by both the physician in charge and radiologists. **(A)** Pretreatment PET-CT image shows that the primary tumor is large, irregular in shape with evident left bronchial compression. The normal esophageal lumen disappears due to extensive thickening of the esophageal wall. **(B)** Posttreatment PET-CT image shows that significant tumor shrinkage provides clear demarcation between primary tumor and the left bronchus. Esophageal lumen reappears. **(C)** Pretreatment PET-CT image presents hypermetabolic characteristic of the primary tumor. **(D)** Subsequent PET-CT image revealed tumor metabolic value reduced to background level.

**Figure 3:**
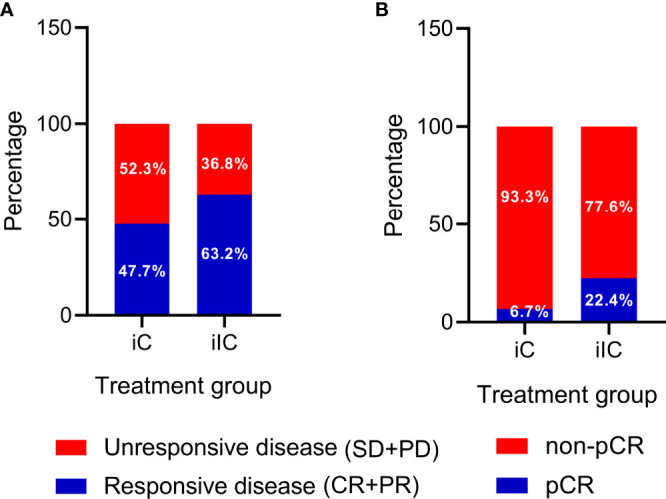
Radiological and pathological responses between induction chemotherapy and induction immunochemotherapy. **(A)** Radiological assessment of tumor responses. Significantly higher responsive disease rate was observed in the iIC cohort. **(B)** Pathological assessment of tumor responses. Significantly higher pCR rate was observed in the iIC cohort. Responsive disease included complete response and partial response. Unresponsive disease included stable disease and progression disease. iC, induction chemotherapy; iIC, induction immunochemotherapy.

Of the 155 patients, six patients (3.9%) achieved radiological CR, 92 patients achieved PR (59.4%), and 45 patients (29%) achieved SD. The ORR and DCR were 63.3% and 92.3%, respectively. A typical case presenting the radiological assessment before and after iIC was shown in **Figure 2**. We categorized patients into radiological responders and radiological non-responders. Responsive disease included CR and PR, whereas unresponsive disease included SD and progression disease. Significant difference in responsive disease rate was observed between the iC cohort and iIC cohort (iIC: 98 of 155, 63.2% vs. iC: 94 of 197, 47.7%, p = 0.004) (**Figure 3A**).

In terms of pathological responses, pCR of the primary tumor was observed in 22.4% (26 of 116) of the patients. mPR was observed in 57.8% (67 of 116) of the patients. R0 resection was achieved in 109 of the 116 patients. **Figure 3** showed both radiological and pathological response rates between iC cohort and iIC cohort. Statistically significant differences in pCR (iIC: 26 of 116, 22.4% vs. iC: 8 of 120, 6.7%, p = 0.001) was revealed (**Figure 3B**). The TRG scores were: TRG 1a (ypT0, 26 of 116, 22.4%), TRG1b (48 of 116, 41.4%), TRG 2 (16 of 116, 13.85%), and TRG 3 (26 of 116, 22.4%). Swimmer plot (**Supplementary Figure 2A**) and waterfall plot (**Supplementary Figure 2B**) were used to depict treatment course and treatment response of the patients. Downstaging of tumor stage was achieved in 111 (of 116, 95.7%) patients. Moreover, downstaging of clinical N stage was achieved in 41% (48 of 116) of patients, whereas 11.2% (13 of 116) of patients had an upstaging in N stage postoperatively.

**Figure 4:**
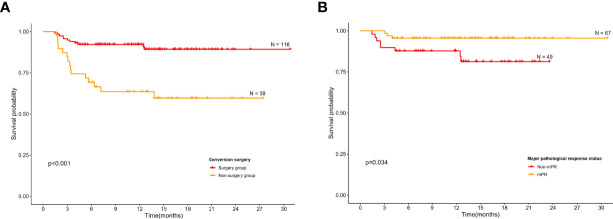
Kaplan–Meier curves for event-free survival. **(A)** Conversion surgery group versus non-surgery group among all patients. **(B)** Event-free survival according to major pathological complete response.

Overall, 12 (of 155; 7.7%) patients had radiological PDs and 10 (of 155; 6.5%) patients died during follow-up. Significantly higher EFS was observed in those who underwent conversion surgery than those in the non-surgery group (**Figure 4A**). Those had a mPR status also demonstrated a significantly higher EFS than the non-mPR cohort (**Figure 4B**), and there is a statistical difference between pCR and non-pCR patients. Further, the survival plot showed a trend of better EFS in surgical candidates who actually received surgery as subsequent treatment than those who declined surgery regardless of their medical fitness upon preoperative evaluation (**Supplementary Figure 3**).

### Expression of PD-L1 of Clinical Specimens

The PD-L1 CPS scores of the surgical candidates were evaluated and compared. No significant association was found among patients with different TRGs (p = 0.206). Moreover, PD-L1 expression did not correlate significantly with both pathological and radiological responses (p = 0.486).

## Discussion

Conversion surgery following iIC for initially unresectable locally advanced ESCC has been reported (8). However, real-world evidence is currently unavailable. The feasibility of immunochemotherapy in the present study was 85.2%, which was comparable to both induction chemoradiotherapy and chemotherapy alone. Moreover, the previously reported conversion rates in induction chemoradiotherapy ranged from 42.6% to 69% (1, 9, 20), and the conversion rates in iC fell between 32% and 65% (1, 10, 21, 22), which were lower than the 74.8% reported in the current study. This finding suggested that induction immunochemotherapy could improve the curative resection rates in patients with initially unresectable ESCC. Furthermore, it was found that conversion surgery could bring about significantly higher EFS than those without conversion surgery (**Figure 4A**). Taken together, induction immunochemotherapy plus conversion surgery may benefit more patients due to its high feasibility and potential survival benefit than the current standard-of-care approach.

Generally, the total grade ≥ 3 TRAEs of iIC incidence was relatively manageable and acceptable. A higher incidence of grade ≥ 3 TRAEs was reported in several studies in which induction therapy was adopted (1, 21). Sugimura et al. reported that in iC, the incidence of grade ≥ 3 neutropenia was 41% and the incidence of lymphopenia was 12% (1), which were higher than those in the current study. The safety profile of the current study was similar to the studies conducted by Cheng et al. (23) and Gu et al. (24), indicating that chemotherapy in combination with immunotherapy may not enhance accumulative toxicities compared with chemotherapy alone. Moreover, the safety profiles of immunochemotherapy were comparable and manageable in both the induction and the neoadjuvant settings. However, immune-related TRAEs such as rash and pruritus were not reported in the chemotherapy and radiotherapy-based cohorts. It was reported that immunotherapy could increase activation of B cells, which further release excessive inflammatory cytokines and thus leads to cutaneous adverse events (25). These results suggested that the safety profile of iIC was comparable to that of standard preoperative treatment for initially unresectable ESCC. Although these studies had heterogenous designs, sample sizes, and ethnic disparities, their consensus results indicated that immunochemotherapy was safe to use in the induction settings for advanced esophageal cancer. Despite this, the severe adverse events in certain individuals could not be neglected. It remains problematic to identify patients in danger of grade ≥ 3 TRAEs in advances. Larger-scale studies are needed to address this issue.

Compared with the iC cohort, intraoperative events in the RICE-retro cohort such as median operative time and blood loss were more favorable. Furthermore, the occurrences of postoperative complications in the RICE-retro cohort were also significantly lower than those in the iC cohort, indicating that the conversion surgery following immunochemotherapy did not bring about more intraoperative or postoperative burdens to both surgeons and patients. It was reported that dense fibrosis in the esophageal mesentery occurred after induction immunochemotherapy, which increased the difficulty of surgery (8). However, in this multicenter, real-world study, despite the formation of scar tissues, we discovered that the significant shrinkage of primary tumor actually lowered the surgical difficulty.

We also evaluated the effectiveness of iIC, which had achieved a promising ORR (63.3%), DCR (92.3%), and pCR (22.4%). The ORR varied from 20.2% to 72% in studies concerning iC plus radiotherapy (1, 21). The ORR derived from RICE-retro cohort falls within the upper range. The current study demonstrated that iIC had a superior radiological response rate over iC alone. The synergistic effect of chemotherapy and immunotherapy has been explored in the molecular level. Research showed that chemotherapy could downregulate coinhibitory molecules such as PD-L1 on the surface of cancer cells (26). Moreover, the combination of ICIs and chemotherapy could synergistically induce antigen-specific immunity and enhance the infiltration of CD8^+^, and CD4^+^FoxP3 T cells to the tumor microenvironment (27). However, the pCR rate of iIC did not have distinct advantage over that of other induction regimens, indicating that local cancer therapy such as radiotherapy, if applicable, may be needed to improve the locoregional therapeutic efficacy.

In terms of the use of immunochemotherapy in the neoadjuvant setting, Li et al. reported that ORR and pCR reached 100% and 56%, respectively, in PALACE-1 (28). Other phase II clinical trials adopting neoadjuvant immunochemotherapy reported that ORR ranged from 66.7% to 85% and pCR ranged from 16.7% to 45.4% (23, 28–36). Compared with these studies, the disease response rate reported by RICE-retro study appeared to be lower than most neoadjuvant immunochemotherapy studies. There are several possible explanations for this result. First, in this real-world study, most participants had more advanced tumor and nodal stages and therefore later clinical stages. Immunotherapy combined with chemotherapy achieved poorer effectiveness in patients with a more advanced pretreatment clinical stage (7). Even so, RICE-retro indicated that 95.7% of patients achieved T downstaging and that more than one-third of the patients achieved N downstaging. Second, the difference in sample sizes between RICE-retro and these clinical trials should be taken in consideration. The number of participants vary from 13 to 56 patients in other clinical trials, whereas 155 patients were included in RICE-retro cohort. Larger sample size may not necessarily guarantee robustness of the conclusion. However, a relatively larger amount of data generated from the multicenter studies could reduce potential bias as well as provide more generalized evidence. Third, the unstandardized pathological assessment such as incomplete specimen sampling may generate false-negative results which cause highly inflated pCR rate. It was noteworthy that the recorded pCR from RICE-retro reached 22.4% after re-evaluating the slides from the enrolled centers according to a standardized protocol. Insufficient information regarding pCR assessment process was provided by different medical centers that investigate the efficacy or effectiveness of iIC; thus, a high pCR rate should be cautiously interpreted.

To the best of our knowledge, this is the first and largest multicenter real-world study investigating the feasibility, effectiveness, and safety profiles of iIC in patients with ESCC. Despite the retrospective nature, the current study provides unique real‐world data that reflected the pragmatic clinical practice differing from the ideal setting of clinical trials. Nonetheless, this study also had several limitations. First, the endoscopic ultrasonography was not applied to all patients in the pretreating assessment of tumor stage because some tumors were too bulky for the endoscope to pass through the esophageal tract. However, similar to that reported by Hashimoto et al., the pathologists observed evidence of tumor regression changes in all layers of the esophageal walls in the resected specimen (37). Second, the effectiveness or safety profiles should be cautiously interpreted due to the implementation of miscellaneous ICIs in our study and their potentially different pharmacodynamics and pharmacokinetics.

Our results supported that conversion surgery following immunochemotherapy is feasible and safe for patients with initially unresectable locally advanced ESCC. Both radiological and pathological response rates were significantly higher in the iIC cohort compared with those in the iC cohort. These findings provide new insight into the role of iIC, further larger-scale studies are needed to establish the standard-of-care use of iIC in the preoperative settings for patients with initially unresectable ESCC.

## Data Availability Statement

All data needed to evaluate the conclusions in the paper are present in the paper and/or the **Supplementary Material**.

## Ethics Statement

The studies involving human participants were reviewed and approved by Institutional Review Board (IRB) of Guangdong Provincial People’s Hospital. The patients/participants provided their written informed consent to participate in this study.

## Author Contributions

SH, HW, and CC: Conceptualization, Data curation, Formal analysis, Validation, Roles/Writing - original draft, Writing - review & editing. MZ, EX, WL, GW, JT, XB, DZ, LX, HYZ, GC, WZ, YT, FX, ZD, ZX, FW, ZH, HZ, XS, ZL, TS, JL, SY, and SX: Methodology, Resources, Writing - review & editing. GQ and JF: Conceptualization, Project administration, Resources, Supervision, Writing - review & editing. All authors contributed to the article and approved the submitted version.

## Funding

This work was supported by a grant from the 2020–2021 Popularization of Science and Technology Innovation Special Project of Guangdong Province of China (2020A1414070007); the Science and Technology Program of Guangzhou, China (201704020107 and 202206010103); the Science and Technology Program of Guangdong, China (210716126901104); and Natural Science Foundation of Guangdong Province (2022A1515012469).

## Conflict of Interest

The authors declare that the research was conducted in the absence of any commercial or financial relationships that could be construed as a potential conflict of interest.

## Publisher’s Note

All claims expressed in this article are solely those of the authors and do not necessarily represent those of their affiliated organizations, or those of the publisher, the editors and the reviewers. Any product that may be evaluated in this article, or claim that may be made by its manufacturer, is not guaranteed or endorsed by the publisher.

## Supplementary Material

Supplementary Figure 1 Association between treatment-related adverse events and clinicopathological information. The clinical heatmap depicts the included individuals’ clinicopathological features and their reported adverse events. Different color blocks were used to classify each clinicopathological features. Darkness of red color represents severity of the treatment-related adverse events.

Supplementary Figure 2 Swimmer plot **(A)** and waterfall plot **(B)**. **(A)** The swimmer plot depicts each patient as one line. C1 represents the first cycle from initiation of the first immunochemotherapy to initiation of the second immunochemotherapy and so on. Various colors and shapes are used to represent the radiological outcomes and treatment-related adverse events. **(B)** Maximum radiological response from baseline. Color blocks represent different radiological outcome per RECIST 1.1.

Supplementary Figure 3 Event-free survival according to willingness to undergo conversion surgery among surgical candidates.
