# Supplementary figures and images for: Conversion Surgery Following Immunochemotherapy in Initially Unresectable Locally Advanced Esophageal Squamous Cell Carcinoma—A Real-World Multicenter Study (RICE-Retro)
Source: Front Immunol. 2022 Jul 13;13:935374. doi: 10.3389/fimmu.2022.935374 (PMC9326168; doi:10.3389/fimmu.2022.935374)

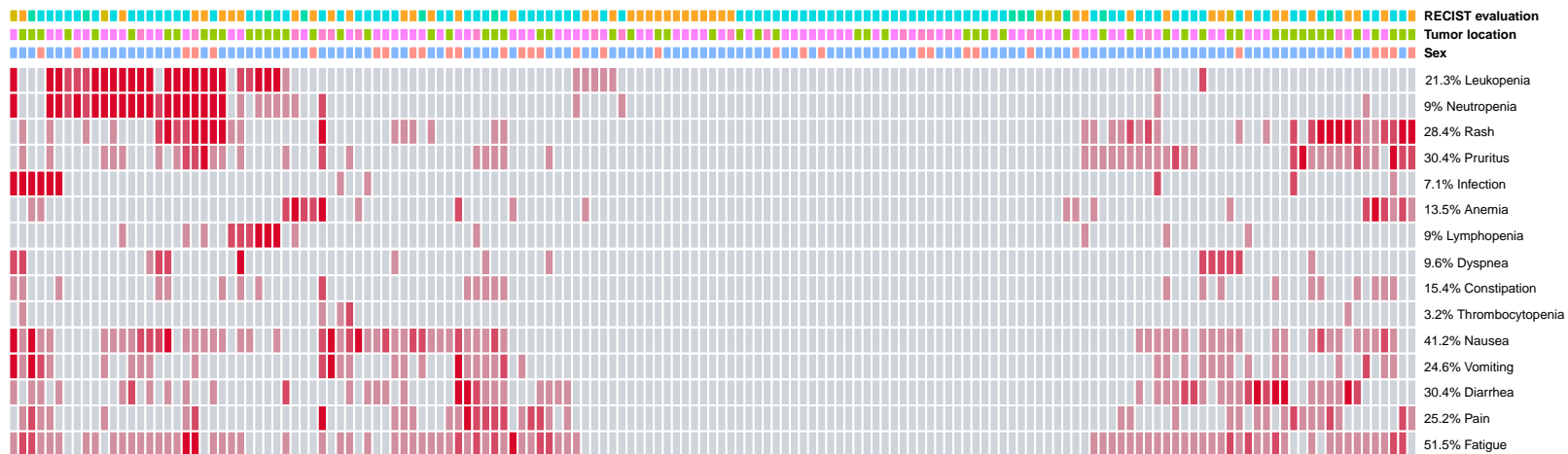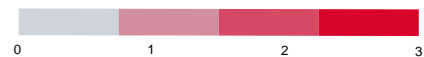

Supplement: Supplementary Figure 1 — Association between treatment-related adverse events and clinicopathological information. The clinical heatmap depicts the included individuals’ clinicopathological features and their reported adverse events. Different color blocks were used to classify each clinicopathological features. Darkness of red color represents severity of the treatment-related adverse events. [file DataSheet_1.pdf]

id

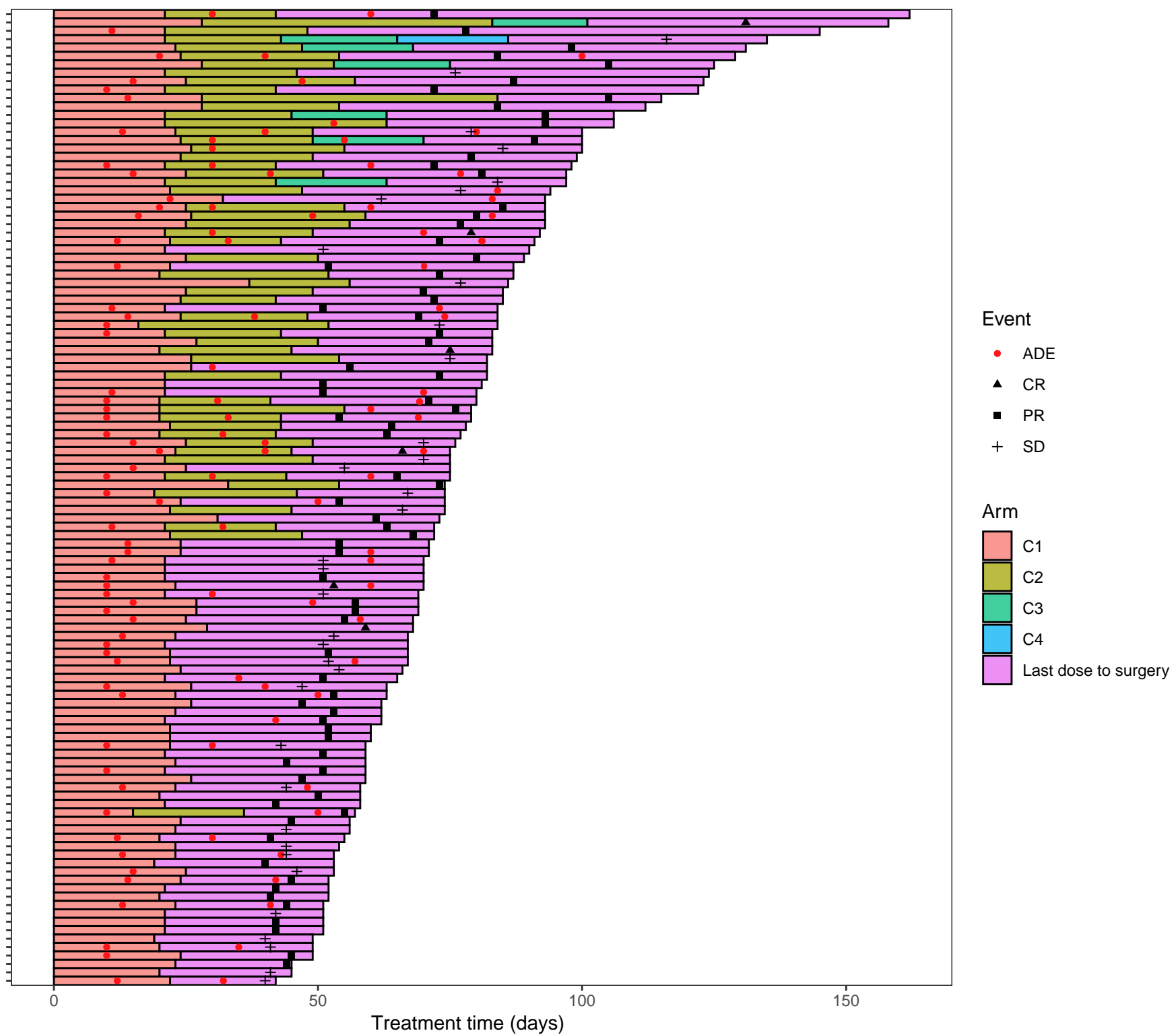

Supplement: Supplementary Figure 2 — Swimmer plot (A) and waterfall plot (B). (A) The swimmer plot depicts each patient as one line. C1 represents the first cycle from initiation of the first immunochemotherapy to initiation of the second immunochemotherapy and so on. Various colors and shapes are used to represent the radiological outcomes and treatment-related adverse events. (B) Maximum radiological response from baseline. Color blocks represent different radiological outcome per RECIST 1.1. [file DataSheet_2.pdf]

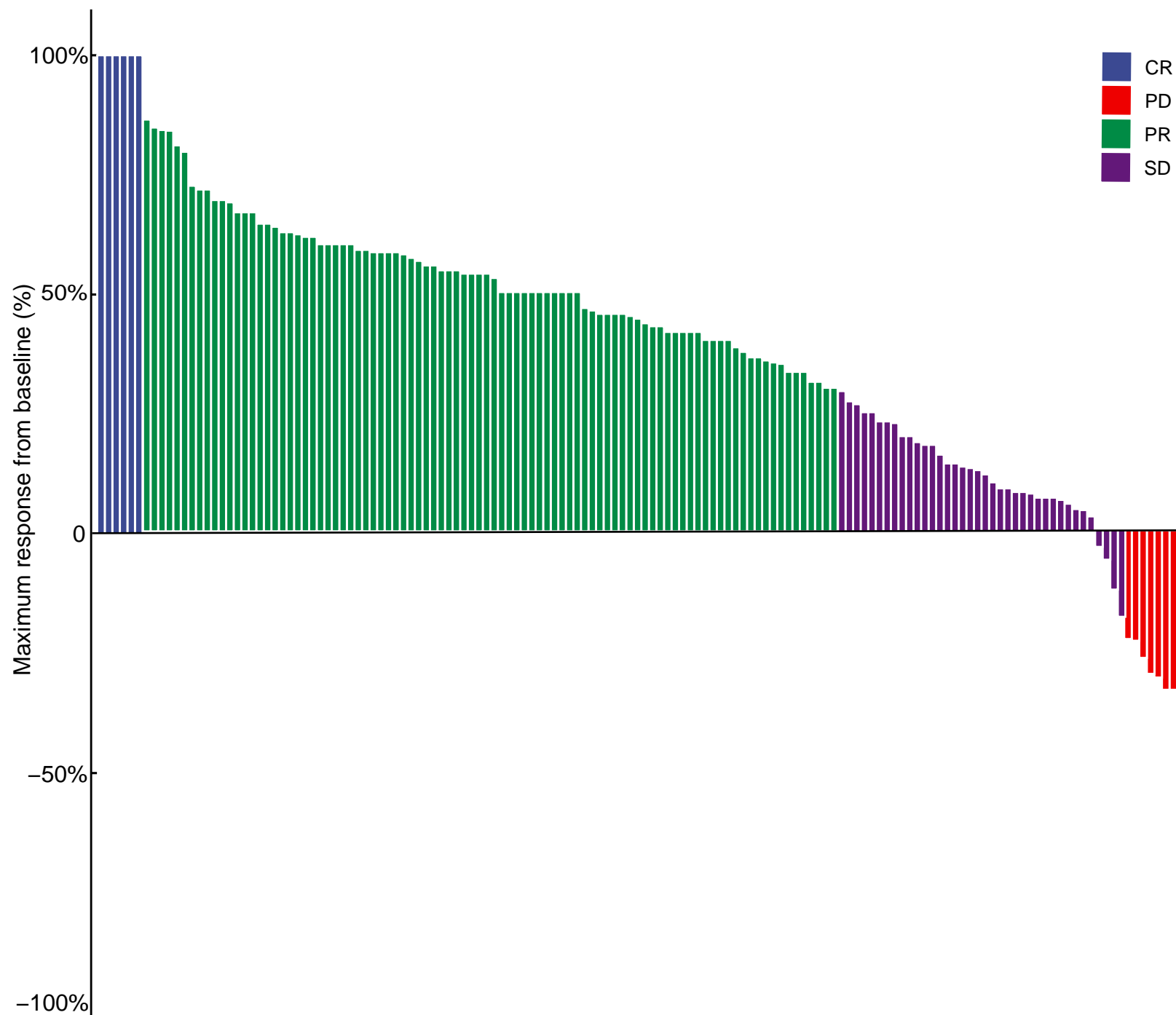

Supplement: Supplementary Figure 3 — Event-free survival according to willingness to undergo conversion surgery among surgical candidates. [file DataSheet_3.pdf]

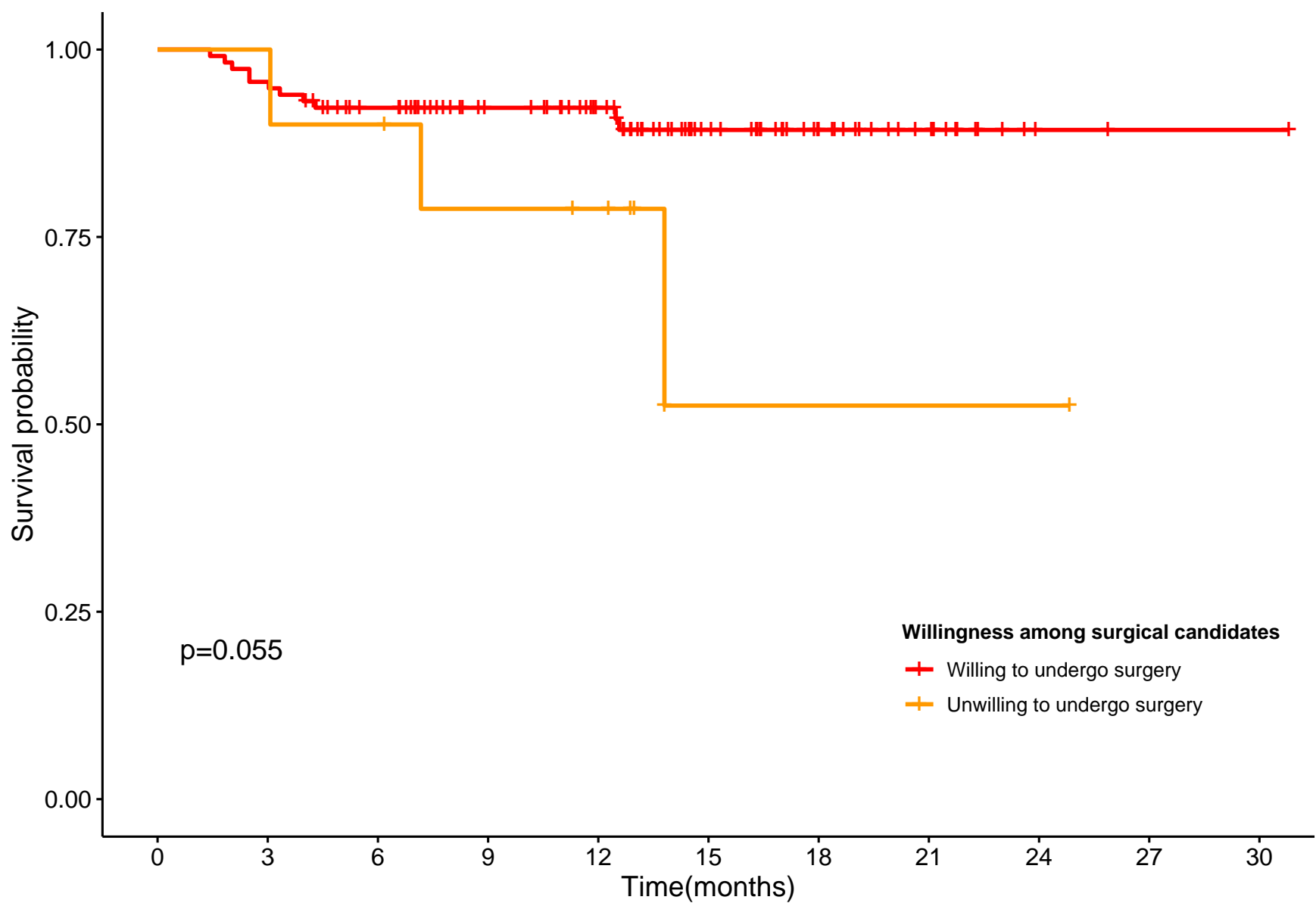

Supplement: Supplementary file 4 [file DataSheet_4.pdf]
