# Supplementary material for: Conversion Surgery Following Immunochemotherapy in Initially Unresectable Locally Advanced Esophageal Squamous Cell Carcinoma—A Real-World Multicenter Study (RICE-Retro)
Source: Front Immunol. 2022 Jul 13;13:935374. doi: 10.3389/fimmu.2022.935374 (PMC9326168; doi:10.3389/fimmu.2022.935374)
Supplement: Supplementary file 5 [file Table_1.docx]

| **Supplementary Table 1. Postoperative events in induction chemotherapy cohort** | |
| --- | --- |
| **Event** | **No. (%)** |
| Heart issues | 0 (0) |
| Pneumonia | 23 (19.2) |
| Atelectasis | 4 (3.3) |
| Pleural effusion | 29 (24.2) |
| Anastomosis fistula | 23 (19.2) |
| Wound infection | 14 (11.7) |
| Hoarseness | 2 (1.7) |
| Hypoxia | 8 (6.7) |
| Dysphagia | 3 (2.5) |
| Hemothorax | 6 (5.0) |
| Chylothorax | 1 (0.8) |
| Mediastinitis | 0 (0) |
| Death | 4 (3.3) |
